# 2,4-Dibromo-6-{(*E*)-[(*R*)-1-phenyl­ethyl]imino­meth­yl}phenol

**DOI:** 10.1107/S1600536809044638

**Published:** 2009-11-21

**Authors:** Dong-Guo Xia, Ya-Fen Ye, Ke-Wei Lei

**Affiliations:** a State Key Laboratory Base of Novel Functional Materials and Preparation Science Institute of solid Materials Chemistry, Faculty of Materials Science and Chemical Engineering, Ningbo University, Ningbo 315211, People’s Republic of China

## Abstract

In the title Schiff base, C_15_H_13_Br_2_NO, the benzene and phenyl rings form a dihedral angle of 75.18 (13)°. The N=C bond length of 1.263 (6) Å is shorter than of the N—C bond [1.476 (5) Å], indicating a double bond. In the crystal, there is some pseudosymmetry. This occurs because most of the two mol­ecules are centrosymmetrically related. The mol­ecular structure is stabilized by intra­molecular O—H⋯N hydrogen bonds.

## Related literature

For photochromism and thermochromism in Schiff base compounds, see: Cohen *et al.* (1964 ▶).
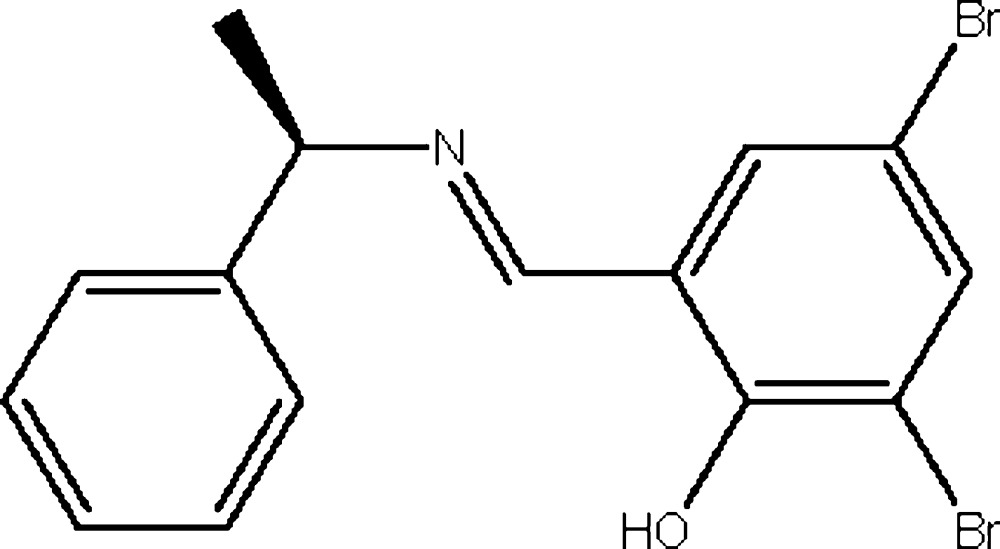


## Experimental

### 

#### Crystal data

C_15_H_13_Br_2_NO
*M* *_r_* = 383.08
Monoclinic, 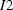

*a* = 15.523 (2) Å
*b* = 9.3533 (12) Å
*c* = 21.527 (4) Å
β = 109.287 (2)°
*V* = 2950.1 (7) Å^3^
*Z* = 8
Mo *K*α radiation
μ = 5.49 mm^−1^
*T* = 296 K
0.38 × 0.31 × 0.26 mm

#### Data collection

Bruker SMART APEXII CCD diffractometer
Absorption correction: multi-scan (*SADABS*; Sheldrick, 2000 ▶) *T*_min_ = 0.158, *T*_max_ = 0.236
12876 measured reflections
6480 independent reflections
4357 reflections with *I* > 2σ(*I*)
*R*_int_ = 0.026

#### Refinement

*R*[*F* ^2^ > 2σ(*F* ^2^)] = 0.036
*wR*(*F* ^2^) = 0.082
*S* = 0.99
6480 reflections
346 parameters
1 restraint
H atoms treated by a mixture of independent and constrained refinement
Δρ_max_ = 0.62 e Å^−3^
Δρ_min_ = −0.50 e Å^−3^
Absolute structure: Flack (1983 ▶), 3189 Friedel pairs
Flack parameter: 0.022 (9)

### 

Data collection: *APEX2* (Bruker, 2007 ▶); cell refinement: *SAINT* (Bruker, 2007 ▶); data reduction: *SAINT*; program(s) used to solve structure: *SHELXS97* (Sheldrick, 2008 ▶); program(s) used to refine structure: *SHELXL97* (Sheldrick, 2008 ▶); molecular graphics: *SHELXTL* (Sheldrick, 2008 ▶); software used to prepare material for publication: *SHELXTL*.

## Supplementary Material

Crystal structure: contains datablocks global, I. DOI: 10.1107/S1600536809044638/bv2125sup1.cif

Structure factors: contains datablocks I. DOI: 10.1107/S1600536809044638/bv2125Isup2.hkl

Additional supplementary materials:  crystallographic information; 3D view; checkCIF report

## Figures and Tables

**Table 1:** Hydrogen-bond geometry (Å, °)

*D*—H⋯*A*	*D*—H	H⋯*A*	*D*⋯*A*	*D*—H⋯*A*
O1—H1⋯N1	0.81	1.89	2.603 (4)	147
O2—H2⋯N2	0.87	1.79	2.558 (5)	147

**Fig. 1.**
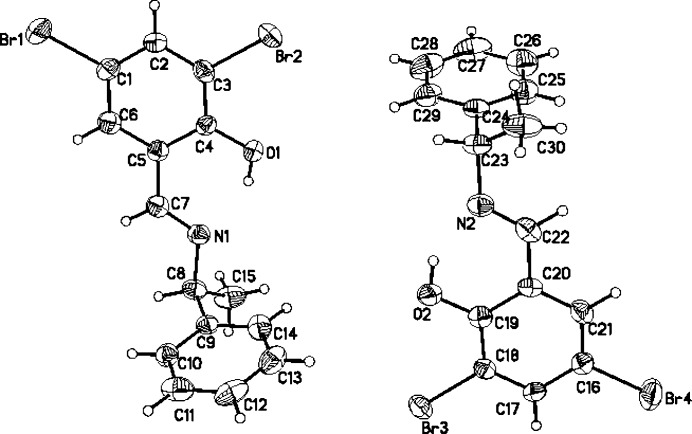
The structure of (I), showing 30% probability displacement ellipsoids and the atom-numbering scheme.

## supplementary crystallographic information

### Comment

Schiff bases have been used extensively as ligands in the field of coordination chemistry. Some of the reasons are that the N atom plays an important role in the formation of metal complexes, and that Schiff base coppounds show photochromism and thermochromism in the solid state by proton transfer from the hydroxyl O atom to the imine N atom (Cohen *et al.*, 1964). Here we report on a new chiral Schiff base(I).

The chiral molecular structures of (I) which are two molecules in the illustrated in Fig. 1. The bond lengths and bond angles in (I) are within normal ranges. The N1–C7 distance of 1.263 (6) Å is a slightly smaller than the distance of N2–C22(1.270 (6)). The C7, N1, C8 and C2, C1, Br1 atoms form a bond angle of 119.3 (4) and 119.5 (3) °, respectively (Table 1). The molecular conformation is stabiized by an intramolecular O–H···N hydrogen bond (Table 2).

### Experimental

*R*-1-phenylethanamine (0.02 mol,2.42 g) and 3,5-dibromo-2-hydroxybenzaldehyde (0.02 mol,5.60 g) were dissolved in ethanol and the solution was refluxed for 4 h. After evaporation, a crude product was recrystallized twice from ethanol to give a pure yellow product. Yield: 83.7%. Calcd.for C_15_H_13_Br_2_NO: C, 47.03; H, 3.42; N, 3.66; Found: C, 46.95; H, 3.49; N, 3.62%.

### Refinement

All H atoms were located from difference Fourier syntheses, H atoms from the C—H groups were placed in geometrically idealized positions and constrained to ride on their parent atoms (C—H = 0.93%A, 0.96%A, 0.97%A;) and *U*_iso_(H) values equal to 1.2 *U*_eq_(C).

### Crystal data

**Table:**

C_15_H_13_Br_2_NO	*F*(000) = 1504
*M**_r_* = 383.08	*D*_x_ = 1.725 Mg m^−^^3^
Monoclinic, *I*2	Mo *K*α radiation, λ = 0.71073 Å
Hall symbol: I 2y	Cell parameters from 3597 reflections
*a* = 15.523 (2) Å	θ = 2.4–23.4°
*b* = 9.3533 (12) Å	µ = 5.49 mm^−^^1^
*c* = 21.527 (4) Å	*T* = 296 K
β = 109.287 (2)°	Block, yellow
*V* = 2950.1 (7) Å^3^	0.38 × 0.31 × 0.26 mm
*Z* = 8	

### Data collection

**Table:**

Bruker SMART APEXII CCD diffractometer	6480 independent reflections
Radiation source: fine-focus sealed tube	4357 reflections with *I* > 2σ(*I*)
graphite	*R*_int_ = 0.026
ω scans	θ_max_ = 27.5°, θ_min_ = 2.0°
Absorption correction: multi-scan (*SADABS*; Sheldrick, 2000)	*h* = −20→20
*T*_min_ = 0.158, *T*_max_ = 0.236	*k* = −12→11
12876 measured reflections	*l* = −27→26

### Refinement

**Table:**

Refinement on *F*^2^	Secondary atom site location: difference Fourier map
Least-squares matrix: full	Hydrogen site location: inferred from neighbouring sites
*R*[*F*^2^ > 2σ(*F*^2^)] = 0.036	H atoms treated by a mixture of independent and constrained refinement
*wR*(*F*^2^) = 0.082	*w* = 1/[σ^2^(*F*_o_^2^) + (0.0192*P*)^2^ + 1.2456*P*] where *P* = (*F*_o_^2^ + 2*F*_c_^2^)/3
*S* = 0.99	(Δ/σ)_max_ = 0.002
6480 reflections	Δρ_max_ = 0.62 e Å^−^^3^
346 parameters	Δρ_min_ = −0.50 e Å^−^^3^
1 restraint	Absolute structure: Flack (1983), 2872 Friedel pairs
Primary atom site location: structure-invariant direct methods	Flack parameter: 0.022 (9)

### Special details

**Table:**

Geometry. All e.s.d.'s (except the e.s.d. in the dihedral angle between two l.s. planes) are estimated using the full covariance matrix. The cell e.s.d.'s are taken into account individually in the estimation of e.s.d.'s in distances, angles and torsion angles; correlations between e.s.d.'s in cell parameters are only used when they are defined by crystal symmetry. An approximate (isotropic) treatment of cell e.s.d.'s is used for estimating e.s.d.'s involving l.s. planes.
Refinement. Refinement of *F*^2^ against ALL reflections. The weighted *R*-factor *wR* and goodness of fit *S* are based on *F*^2^, conventional *R*-factors *R* are based on *F*, with *F* set to zero for negative *F*^2^. The threshold expression of *F*^2^ > σ(*F*^2^) is used only for calculating *R*-factors(gt) *etc*. and is not relevant to the choice of reflections for refinement. *R*-factors based on *F*^2^ are statistically about twice as large as those based on *F*, and *R*- factors based on ALL data will be even larger.

### Fractional atomic coordinates and isotropic or equivalent isotropic displacement parameters (Å^2^)

**Table:**

	*x*	*y*	*z*	*U*_iso_*/*U*_eq_	
Br1	0.37953 (5)	−0.48288 (5)	1.03409 (3)	0.0857 (2)	
Br2	0.34338 (4)	0.04283 (6)	1.15258 (2)	0.07989 (19)	
Br3	0.16026 (3)	0.83627 (6)	0.83602 (2)	0.06812 (15)	
Br4	0.11106 (5)	1.32687 (6)	0.97217 (3)	0.0987 (2)	
N1	0.3623 (2)	0.1446 (4)	0.90617 (16)	0.0519 (9)	
N2	0.1352 (2)	0.6719 (4)	1.07323 (16)	0.0545 (9)	
O1	0.3584 (2)	0.1594 (3)	1.02596 (14)	0.0596 (9)	
H1	0.3621 (3)	0.1891 (19)	0.992 (2)	0.089*	
O2	0.1494 (2)	0.6885 (3)	0.95843 (14)	0.0616 (9)	
H2	0.1469 (3)	0.649 (2)	0.994 (2)	0.092*	
C1	0.3775 (3)	−0.2791 (5)	1.0328 (2)	0.0527 (11)	
C2	0.3679 (3)	−0.2060 (5)	1.0852 (2)	0.0492 (11)	
H2B	0.3653	−0.2546	1.1222	0.059*	
C3	0.3622 (3)	−0.0595 (5)	1.08201 (19)	0.0512 (11)	
C4	0.3661 (3)	0.0165 (5)	1.02755 (18)	0.0434 (9)	
C5	0.3760 (3)	−0.0612 (5)	0.97431 (19)	0.0459 (10)	
C6	0.3816 (3)	−0.2091 (5)	0.9777 (2)	0.0540 (12)	
H6A	0.3881	−0.2610	0.9427	0.065*	
C7	0.3746 (3)	0.0115 (5)	0.9143 (2)	0.0515 (10)	
H7A	0.3832	−0.0423	0.8805	0.062*	
C8	0.3566 (3)	0.2086 (5)	0.84227 (19)	0.0549 (11)	
H8A	0.3705	0.1353	0.8144	0.066*	
C9	0.2603 (3)	0.2606 (4)	0.80995 (18)	0.0448 (10)	
C10	0.2032 (3)	0.1924 (5)	0.7561 (2)	0.0555 (11)	
H10A	0.2249	0.1156	0.7382	0.067*	
C11	0.1137 (3)	0.2351 (7)	0.7277 (2)	0.0750 (15)	
H11A	0.0756	0.1862	0.6914	0.090*	
C12	0.0810 (3)	0.3481 (7)	0.7525 (3)	0.0783 (16)	
H12A	0.0211	0.3784	0.7326	0.094*	
C13	0.1371 (4)	0.4176 (6)	0.8071 (3)	0.0737 (15)	
H13A	0.1145	0.4935	0.8250	0.088*	
C14	0.2261 (3)	0.3758 (5)	0.8354 (2)	0.0601 (12)	
H14A	0.2639	0.4249	0.8717	0.072*	
C15	0.4278 (3)	0.3274 (6)	0.8551 (2)	0.0783 (15)	
H15A	0.4876	0.2875	0.8750	0.117*	
H15B	0.4164	0.3970	0.8843	0.117*	
H15C	0.4242	0.3724	0.8143	0.117*	
C16	0.1200 (3)	1.1252 (5)	0.9680 (2)	0.0541 (11)	
C17	0.1326 (2)	1.0647 (5)	0.91308 (18)	0.0465 (9)	
H17A	0.1352	1.1218	0.8784	0.056*	
C18	0.1413 (3)	0.9194 (5)	0.91066 (18)	0.0447 (10)	
C19	0.1391 (2)	0.8311 (6)	0.96208 (17)	0.0460 (9)	
C20	0.1247 (3)	0.8938 (5)	1.01712 (19)	0.0468 (10)	
C21	0.1163 (3)	1.0434 (5)	1.01940 (19)	0.0553 (12)	
H21A	0.1082	1.0864	1.0560	0.066*	
C22	0.1220 (3)	0.8060 (6)	1.0725 (2)	0.0591 (13)	
H22A	0.1104	0.8490	1.1079	0.071*	
C23	0.1351 (3)	0.5817 (5)	1.12971 (19)	0.0582 (12)	
H23A	0.1198	0.4849	1.1121	0.070*	
C24	0.2320 (3)	0.5733 (5)	1.17797 (19)	0.0508 (10)	
C25	0.2650 (3)	0.6751 (6)	1.2257 (2)	0.0672 (13)	
H25A	0.2273	0.7496	1.2294	0.081*	
C26	0.3544 (4)	0.6682 (7)	1.2688 (3)	0.0884 (18)	
H26A	0.3766	0.7378	1.3010	0.106*	
C27	0.4104 (4)	0.5554 (9)	1.2630 (3)	0.094 (2)	
H27A	0.4697	0.5475	1.2920	0.113*	
C28	0.3767 (4)	0.4568 (8)	1.2141 (3)	0.0921 (19)	
H28A	0.4137	0.3824	1.2092	0.111*	
C29	0.2892 (3)	0.4672 (6)	1.1726 (2)	0.0691 (14)	
H29A	0.2678	0.3995	1.1394	0.083*	
C30	0.0640 (3)	0.6210 (7)	1.1599 (2)	0.0813 (16)	
H30A	0.0051	0.6225	1.1264	0.122*	
H30B	0.0774	0.7139	1.1797	0.122*	
H30C	0.0641	0.5519	1.1929	0.122*	

### Atomic displacement parameters (Å^2^)

**Table:**

	*U*^11^	*U*^22^	*U*^33^	*U*^12^	*U*^13^	*U*^23^
Br1	0.1247 (5)	0.0516 (3)	0.0857 (4)	0.0224 (3)	0.0414 (4)	0.0129 (3)
Br2	0.1140 (4)	0.0779 (4)	0.0438 (2)	0.0113 (3)	0.0207 (2)	−0.0087 (3)
Br3	0.0959 (4)	0.0645 (3)	0.0470 (2)	−0.0069 (3)	0.0277 (2)	−0.0083 (3)
Br4	0.1556 (6)	0.0524 (3)	0.1062 (4)	0.0174 (4)	0.0676 (4)	0.0002 (4)
N1	0.055 (2)	0.055 (3)	0.0459 (19)	−0.0056 (18)	0.0166 (16)	0.0031 (18)
N2	0.050 (2)	0.061 (3)	0.049 (2)	−0.0049 (18)	0.0103 (17)	0.0050 (18)
O1	0.083 (2)	0.0407 (18)	0.0500 (18)	0.0035 (15)	0.0152 (16)	−0.0009 (14)
O2	0.080 (2)	0.050 (2)	0.0496 (18)	−0.0038 (16)	0.0148 (16)	0.0040 (15)
C1	0.048 (3)	0.047 (3)	0.059 (3)	0.009 (2)	0.013 (2)	0.008 (2)
C2	0.049 (3)	0.053 (3)	0.044 (2)	0.003 (2)	0.0131 (19)	0.0082 (19)
C3	0.048 (3)	0.059 (3)	0.040 (2)	0.001 (2)	0.006 (2)	−0.009 (2)
C4	0.039 (2)	0.039 (2)	0.044 (2)	−0.0001 (19)	0.0036 (17)	0.0030 (19)
C5	0.040 (2)	0.048 (3)	0.047 (2)	0.005 (2)	0.0122 (19)	0.003 (2)
C6	0.050 (3)	0.062 (3)	0.053 (3)	0.014 (2)	0.021 (2)	0.001 (2)
C7	0.045 (2)	0.059 (3)	0.051 (2)	0.006 (2)	0.0177 (19)	0.000 (2)
C8	0.057 (3)	0.067 (3)	0.047 (2)	−0.001 (2)	0.025 (2)	0.008 (2)
C9	0.050 (3)	0.047 (2)	0.042 (2)	−0.003 (2)	0.022 (2)	0.0084 (19)
C10	0.068 (3)	0.049 (3)	0.056 (3)	0.002 (2)	0.028 (2)	0.004 (2)
C11	0.062 (3)	0.089 (4)	0.065 (3)	−0.019 (3)	0.008 (3)	0.017 (3)
C12	0.060 (3)	0.077 (4)	0.098 (4)	0.019 (3)	0.027 (3)	0.036 (4)
C13	0.081 (4)	0.058 (3)	0.096 (4)	0.009 (3)	0.049 (3)	0.015 (3)
C14	0.062 (3)	0.058 (3)	0.066 (3)	−0.007 (2)	0.029 (2)	−0.003 (2)
C15	0.056 (3)	0.095 (4)	0.083 (3)	−0.011 (3)	0.022 (2)	0.032 (3)
C16	0.062 (3)	0.051 (3)	0.052 (3)	0.006 (2)	0.022 (2)	0.003 (2)
C17	0.048 (2)	0.045 (2)	0.043 (2)	−0.001 (2)	0.0105 (18)	0.008 (2)
C18	0.041 (2)	0.053 (3)	0.039 (2)	−0.0018 (19)	0.0110 (19)	−0.0010 (19)
C19	0.034 (2)	0.052 (3)	0.046 (2)	−0.008 (2)	0.0057 (17)	−0.001 (2)
C20	0.041 (2)	0.060 (3)	0.038 (2)	0.0021 (19)	0.0121 (18)	0.011 (2)
C21	0.055 (3)	0.067 (3)	0.048 (2)	0.002 (2)	0.023 (2)	−0.005 (2)
C22	0.054 (3)	0.078 (4)	0.046 (2)	−0.002 (3)	0.019 (2)	−0.007 (2)
C23	0.060 (3)	0.065 (3)	0.045 (2)	−0.009 (2)	0.011 (2)	0.013 (2)
C24	0.049 (2)	0.061 (3)	0.044 (2)	−0.003 (2)	0.0170 (18)	0.015 (2)
C25	0.057 (3)	0.074 (3)	0.067 (3)	0.005 (3)	0.015 (2)	0.007 (3)
C26	0.088 (5)	0.093 (5)	0.071 (4)	−0.026 (4)	0.008 (3)	−0.003 (3)
C27	0.046 (3)	0.124 (6)	0.099 (4)	−0.005 (4)	0.006 (3)	0.044 (5)
C28	0.066 (4)	0.103 (5)	0.116 (5)	0.030 (4)	0.042 (4)	0.044 (4)
C29	0.070 (3)	0.073 (4)	0.071 (3)	0.013 (3)	0.032 (3)	0.020 (3)
C30	0.051 (3)	0.124 (5)	0.068 (3)	0.000 (3)	0.018 (2)	0.037 (3)

### Geometric parameters (Å, °)

**Table:**

Br1—C1	1.907 (5)	C13—C14	1.370 (7)
Br2—C3	1.898 (4)	C13—H13A	0.9300
Br3—C18	1.894 (4)	C14—H14A	0.9300
Br4—C16	1.896 (5)	C15—H15A	0.9600
N1—C7	1.263 (6)	C15—H15B	0.9600
N1—C8	1.476 (5)	C15—H15C	0.9600
N2—C22	1.270 (6)	C16—C21	1.362 (6)
N2—C23	1.480 (5)	C16—C17	1.381 (6)
O1—C4	1.342 (5)	C17—C18	1.369 (6)
O1—H1	0.8066	C17—H17A	0.9300
O2—C19	1.348 (6)	C18—C19	1.390 (6)
O2—H2	0.8662	C19—C20	1.405 (6)
C1—C2	1.368 (6)	C20—C21	1.408 (7)
C1—C6	1.374 (6)	C20—C22	1.460 (6)
C2—C3	1.373 (6)	C21—H21A	0.9300
C2—H2B	0.9300	C22—H22A	0.9300
C3—C4	1.389 (6)	C23—C30	1.500 (6)
C4—C5	1.408 (6)	C23—C24	1.521 (6)
C5—C6	1.386 (6)	C23—H23A	0.9800
C5—C7	1.453 (6)	C24—C29	1.362 (6)
C6—H6A	0.9300	C24—C25	1.370 (6)
C7—H7A	0.9300	C25—C26	1.392 (7)
C8—C9	1.507 (6)	C25—H25A	0.9300
C8—C15	1.527 (6)	C26—C27	1.399 (9)
C8—H8A	0.9800	C26—H26A	0.9300
C9—C10	1.363 (5)	C27—C28	1.366 (9)
C9—C14	1.390 (6)	C27—H27A	0.9300
C10—C11	1.379 (6)	C28—C29	1.360 (7)
C10—H10A	0.9300	C28—H28A	0.9300
C11—C12	1.355 (8)	C29—H29A	0.9300
C11—H11A	0.9300	C30—H30A	0.9600
C12—C13	1.373 (7)	C30—H30B	0.9600
C12—H12A	0.9300	C30—H30C	0.9600
			
C7—N1—C8	119.3 (4)	H15A—C15—H15C	109.5
C22—N2—C23	121.9 (4)	H15B—C15—H15C	109.5
C4—O1—H1	109.5	C21—C16—C17	121.4 (4)
C19—O2—H2	109.5	C21—C16—Br4	119.8 (3)
C2—C1—C6	121.5 (4)	C17—C16—Br4	118.8 (3)
C2—C1—Br1	119.5 (3)	C18—C17—C16	118.8 (4)
C6—C1—Br1	118.9 (4)	C18—C17—H17A	120.6
C1—C2—C3	118.7 (4)	C16—C17—H17A	120.6
C1—C2—H2B	120.7	C17—C18—C19	122.1 (4)
C3—C2—H2B	120.7	C17—C18—Br3	119.0 (3)
C2—C3—C4	122.2 (4)	C19—C18—Br3	118.9 (3)
C2—C3—Br2	119.2 (4)	O2—C19—C18	120.4 (4)
C4—C3—Br2	118.6 (3)	O2—C19—C20	121.1 (4)
O1—C4—C3	120.2 (4)	C18—C19—C20	118.4 (5)
O1—C4—C5	121.8 (4)	C19—C20—C21	119.1 (4)
C3—C4—C5	118.0 (4)	C19—C20—C22	120.6 (4)
C6—C5—C4	119.6 (4)	C21—C20—C22	120.2 (4)
C6—C5—C7	119.7 (4)	C16—C21—C20	120.0 (4)
C4—C5—C7	120.6 (4)	C16—C21—H21A	120.0
C1—C6—C5	120.0 (4)	C20—C21—H21A	120.0
C1—C6—H6A	120.0	N2—C22—C20	121.1 (4)
C5—C6—H6A	120.0	N2—C22—H22A	119.4
N1—C7—C5	122.8 (4)	C20—C22—H22A	119.4
N1—C7—H7A	118.6	N2—C23—C30	114.6 (4)
C5—C7—H7A	118.6	N2—C23—C24	108.8 (3)
N1—C8—C9	107.8 (3)	C30—C23—C24	114.8 (4)
N1—C8—C15	108.0 (3)	N2—C23—H23A	106.0
C9—C8—C15	113.4 (4)	C30—C23—H23A	106.0
N1—C8—H8A	109.2	C24—C23—H23A	106.0
C9—C8—H8A	109.2	C29—C24—C25	118.5 (4)
C15—C8—H8A	109.2	C29—C24—C23	120.3 (5)
C10—C9—C14	118.2 (4)	C25—C24—C23	121.1 (4)
C10—C9—C8	120.6 (4)	C24—C25—C26	120.8 (5)
C14—C9—C8	121.2 (4)	C24—C25—H25A	119.6
C9—C10—C11	121.2 (4)	C26—C25—H25A	119.6
C9—C10—H10A	119.4	C25—C26—C27	119.1 (5)
C11—C10—H10A	119.4	C25—C26—H26A	120.4
C12—C11—C10	120.3 (5)	C27—C26—H26A	120.4
C12—C11—H11A	119.8	C28—C27—C26	119.2 (5)
C10—C11—H11A	119.8	C28—C27—H27A	120.4
C11—C12—C13	119.5 (5)	C26—C27—H27A	120.4
C11—C12—H12A	120.3	C29—C28—C27	120.2 (6)
C13—C12—H12A	120.3	C29—C28—H28A	119.9
C14—C13—C12	120.4 (5)	C27—C28—H28A	119.9
C14—C13—H13A	119.8	C28—C29—C24	122.2 (6)
C12—C13—H13A	119.8	C28—C29—H29A	118.9
C13—C14—C9	120.4 (4)	C24—C29—H29A	118.9
C13—C14—H14A	119.8	C23—C30—H30A	109.5
C9—C14—H14A	119.8	C23—C30—H30B	109.5
C8—C15—H15A	109.5	H30A—C30—H30B	109.5
C8—C15—H15B	109.5	C23—C30—H30C	109.5
H15A—C15—H15B	109.5	H30A—C30—H30C	109.5
C8—C15—H15C	109.5	H30B—C30—H30C	109.5

### Hydrogen-bond geometry (Å, °)

**Table:**

*D*—H···*A*	*D*—H	H···*A*	*D*···*A*	*D*—H···*A*
O1—H1···N1	0.81	1.89	2.603 (4)	147
O2—H2···N2	0.87	1.79	2.558 (5)	147
